# Calcium imaging of sleep–wake related neuronal activity in the dorsal pons

**DOI:** 10.1038/ncomms10763

**Published:** 2016-02-25

**Authors:** Julia Cox, Lucas Pinto, Yang Dan

**Affiliations:** 1 Division of Neurobiology, Department of Molecular and Cell Biology, Helen Wills Neuroscience Institute, Howard Hughes Medical Institute, University of California, 230 Li Ka Shing Center, Berkeley, California 94720, USA

## Abstract

The dorsal pons has long been implicated in the generation of rapid eye movement (REM) sleep, but the underlying circuit mechanisms remain poorly understood. Using cell-type-specific microendoscopic Ca^2+^ imaging in and near the laterodorsal tegmental nucleus, we found that many glutamatergic neurons are maximally active during REM sleep (REM-max), while the majority of GABAergic neurons are maximally active during wakefulness (wake-max). Furthermore, the activity of glutamatergic neurons exhibits a medio-lateral spatial gradient, with medially located neurons more selectively active during REM sleep.

## Abstract (web summary)

Dreaming occurs in REM sleep, yet the neural mechanisms involved in generating it are not understood. Here Cox and colleagues show that glutamatergic neurons in the dorsal pons are activated most during transition to REM sleep while GABAergic neurons are more active during waking state.

## Graphical abstract

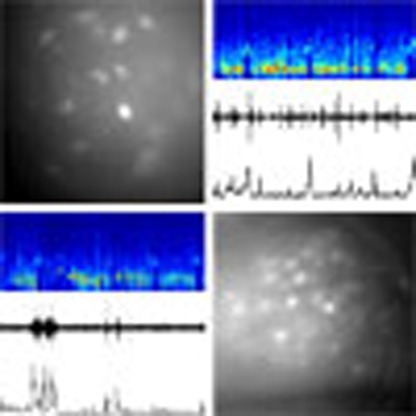

Rapid eye movement (REM) sleep is characterized by desynchronized electroencephalogram (EEG) associated with activated brain state and a lack of electromyogram (EMG) activity indicating skeletal muscle atonia^1^,^2^,^3^. Since the discovery of REM sleep and its associated dreaming, the underlying neural mechanisms have been investigated extensively^4^,^5^. Transection studies showed that the brainstem is necessary and sufficient for REM sleep generation^3^, and subsequent lesion and pharmacological manipulations demonstrated the particular importance of the dorsal pons^6^,^7^,^8^,^9^,^10^,^11^,^12^,^13^,^14^. In addition, many neurons in the dorsal pons express the immediate early gene *c-fos* following deprivation-induced REM sleep rebound^15^,^16^,^17^, indicating that they are active during REM sleep.

There are multiple cell types in the dorsal pons, including cholinergic, monoaminergic, GABAergic and glutamatergic neurons. During REM sleep, cholinergic neurons fire at high rates^18^,^19^, while monoaminergic neurons are virtually silent^20^. The activity of glutamatergic and GABAergic neurons also changes with brain state^16^,^17^,^18^,^19^,^21^,^22^, and these neurons are thought to play important roles in REM sleep regulation^9^,^10^,^12^,^23^. However, compared with cholinergic and monoaminergic neurons, their activity patterns are much more diverse^18^, and their functions in sleep–wake control are less well understood. An important unresolved question is whether neurons with different functional properties are spatially intermingled or segregated into different subregions of the dorsal pons.

To further characterize the sleep–wake activity of glutamatergic and GABAergic neurons and their spatial organization, we performed cellular-resolution Ca^2+^ imaging from the dorsal pontine region in and near the lateral dorsal tegmental nucleus (LDT) in freely moving mice as they transitioned naturally through the sleep–wake cycle^24^,^25^. Using a genetically encoded Ca^2+^ indicator, we show that the activity of both GABAergic and glutamatergic neurons is strongly modulated by brain state. Many glutamatergic neurons are maximally active during REM sleep, and their REM selectivity is organized along the mediolateral axis in the dorsal pons.

## Results

**Figure 1:**
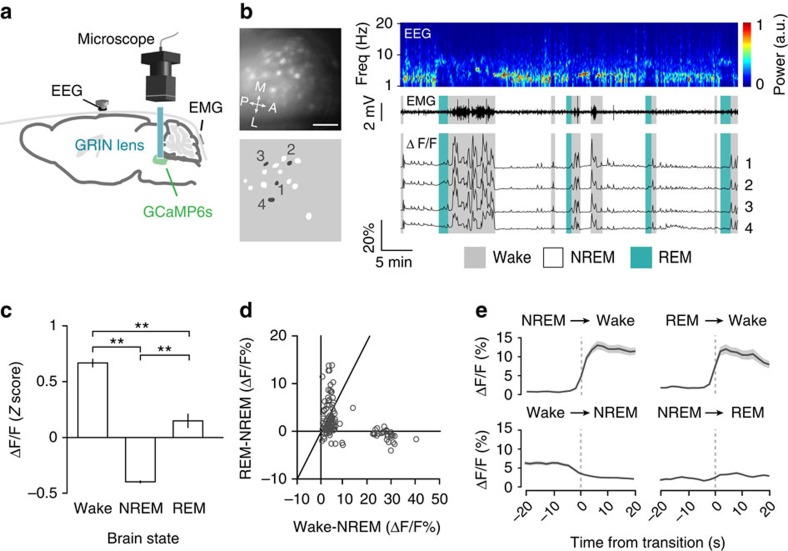
Calcium imaging from GABAergic neurons in the dorsal pons. (**a**) Schematic of microendoscopic Ca^2+^ imaging and brain state recording. (**b**) An example imaging session from a *GAD2-IRES-Cre* mouse. Left panels: imaging field (upper) and identified ROIs corresponding to neuronal somas (lower). Grey masks and numbers indicate ROIs whose ΔF/F traces are plotted on the right. Scale bar, 100 μm. Right panels: EEG power spectrogram, EMG trace and ΔF/F traces. Brain states are indicated by coloured shading. Grey, wake; white, NREM; blue, REM (**c**) *Z*-scored ΔF/F activity during wakefulness, NREM and REM sleep averaged across all GABAergic neurons imaged in six mice (*n*=130). *F*_2,129_=160.7, *P*=4.7 × 10^−46^, one-way repeated measures ANOVA; **P<0.01, two-tailed Tukey's *post hoc* comparison. (**d**) Difference between REM and NREM activity versus difference between wake and NREM activity. Each symbol represents one neuron (*n*=130). Diagonal line: equal activity between wake and REM states. The majority of GABAergic neurons were more active during wake than REM states (below diagonal). (**e**) ΔF/F activity at brain state transitions, averaged across all GABAergic neurons. Grey shading, ±s.e.m.

To image each cell type, we injected adeno-associated virus (AAV) into the dorsal pons (in or near the LDT) of *GAD2-IRES*- or *VGLUT2-IRES-Cre* mice for Cre-inducible expression of the Ca^2+^ indicator GCaMP6s (ref. 24). Calcium imaging was performed through a gradient refractive index (GRIN) lens coupled to a miniaturized integrated fluorescence microscope (Fig. 1a, Supplementary Fig. 1)^25^. Each imaging session lasted between 60 and 152 min (median 88), encompassing multiple cycles of wake, REM and non-REM (NREM) states that were classified based on EEG and EMG recordings (Methods section).

### Sleep–wake activity of GABAergic neurons

Both GABAergic and glutamatergic cells exhibited brain-state-dependent Ca^2+^ activity (Supplementary Movies 1 and 2). As shown in an example imaging session from a *GAD2-IRES-Cre* mouse (Fig. 1b), each field of view contained multiple GABAergic neurons. These neurons showed large Ca^2+^ transients during wakefulness, especially during periods of high EMG activity. In many cases, small Ca^2+^ transients were observed during sleep as well, usually with lower amplitudes and rates during NREM than REM sleep. All GABAergic neurons imaged from 6 *GAD2-IRES-Cre* mice (*n*=130) were significantly modulated by brain state (*P*<0.001, one-way ANOVA), and they were significantly more active during wakefulness and/or REM sleep than NREM sleep (Fig. 1b–d, *P*<0.05, two-tailed Tukey's *post hoc* test). The majority of these neurons (93/130, 71.5%) was significantly more active during wakefulness than REM sleep (wake-max; *P*<0.05, two-tailed Tukey's *post hoc* test), but a small percentage (21/130, 16.2%) was most active during REM sleep (REM-max; Fig. 1c,d). The activity of the GABAergic neurons began to increase 3–5 s before the transition from NREM or REM to wake states and began to decrease 5–10 s before the wake-to-NREM transition (Fig. 1e). Although the activity of some neurons also increased at the NREM-to-REM transition, the magnitude was much lower than at the sleep-to-wake transitions (Supplementary Fig. 2).

### Sleep–wake activity of glutamatergic neurons

**Figure 2:**
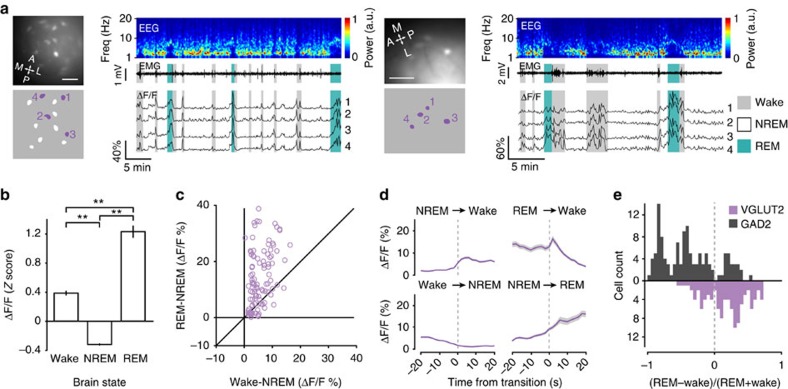
Calcium imaging from glutamatergic neurons in the dorsal pons. (**a**) Example imaging sessions from two *VGLUT2-IRES-Cre* mice. Shown in each example are imaging field, identified ROIs, EEG power spectrogram, EMG trace and ΔF/F traces of example ROIs across brain states (coloured shading). Grey, wake; white: NREM; blue: REM. Scale bars, 100 μm. (**b**) *Z*-scored ΔF/F activity during wakefulness, NREM sleep and REM sleep averaged across all glutamatergic neurons imaged in seven mice (*n*=104). *F*_2,103_=234.6, *P*=8.0 × 10^−54^, one-way repeated measures ANOVA; **P<0.01, two-tailed Tukey's *post hoc* comparisons. (**c**) Difference between REM and NREM activity versus difference between wake and NREM activity. Each symbol represents one neuron (*n*=104). Diagonal line: equal activity between wake and REM states. Most of the glutamatergic neurons were above the diagonal. (**d**) ΔF/F activity at brain state transitions averaged across all glutamatergic neurons. Grey shading, ±s.e.m. (**e**) Distributions of relative REM versus wake activity of GABAergic and glutamatergic neurons (>0, more active during REM than wakefulness).

Figure 2a shows two example imaging sessions from glutamatergic neurons in the dorsal pons. All of these neurons (*n*=104, imaged from 7 *VGLUT2-IRES-Cre* mice) were significantly modulated by brain state (*P*<0.01, one-way ANOVA), and the great majority (103/104, 99.0%) was more active during wakefulness than NREM sleep (Fig. 2a–c; *P*<0.05, two-tailed Tukey's *post hoc* test). However, unlike the GABAergic neurons, the majority of glutamatergic cells (71/104, 68.3%) showed significantly higher activity during REM sleep than wakefulness (REM-max, *P*<0.05, two-tailed Tukey's *post hoc* test; Fig. 2c), and only a small percentage (17/104, 16.3%) was wake-max. The Ca^2+^ levels of glutamatergic neurons increased at NREM-to-wake transitions and decreased at wake-to-NREM transitions (Fig. 2d). Interestingly, at REM-to-wake transitions, the activity increased only transiently, followed by a strong, prolonged decrease (Fig. 2d, upper right, Supplementary Fig. 2). Glutamatergic neurons also showed a pronounced increase in activity at NREM-to-REM transitions, which was significantly greater than that of GABAergic neurons (*Z*=–8.23, *P=*1.9 × 10^−16^, two-tailed Wilcoxon rank-sum test, *n*=208).

Note that for both glutamatergic and GABAergic neurons, the cells imaged simultaneously in the same field of view showed highly correlated activity (Figs 1b and 2a). To test whether this is caused by contamination of the somatic Ca^2+^ signals by out-of-focus neuropil fluorescence (even though neuropil fluorescence was subtracted from the measured somatic signals, Methods section, Supplementary Fig. 3b), in a small number of mice, we performed two-photon Ca^2+^ imaging through the GRIN lens, which decreases neuropil contamination due to better axial resolution^26^. We still found highly correlated activity among simultaneously imaged neurons (Supplementary Fig. 3), suggesting that it is not an artifact of neuropil contamination.

Although brain-state-dependent activity of cholinergic neurons has been well characterized in previous electrophysiological studies^18^,^19^, we also imaged from a small number of these neurons in *ChAT-IRES-Cre* mice. In contrast to the GABAergic and glutamatergic neurons, cholinergic neurons in the LDT were similarly active during wakefulness and REM sleep (Supplementary Fig. 4).

### Spatial organization of glutamatergic sleep–wake activity

**Figure 3:**
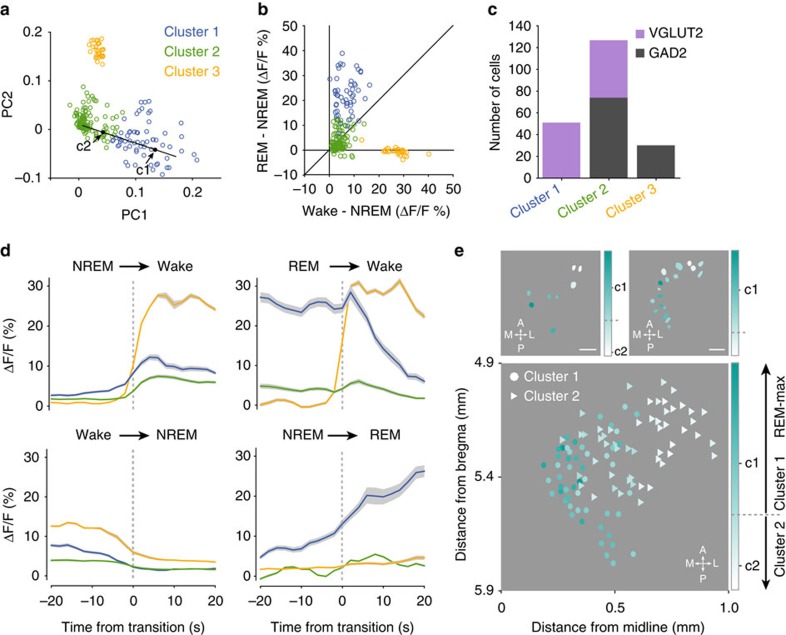
Spatial distributions of different functional types of glutamatergic neurons. (**a**) Scatter plot of both glutamatergic and GABAergic neurons in the space spanned by the first two principal components (PC1 versus PC2) of ΔF/F activity at brain state transitions (*n*=220). The cluster identity determined with k-means clustering is indicated by colour. The centroids of Clusters 1 and 2 are marked as c1 and c2. (**b**) REM–NREM versus wake–NREM activity of all glutamatergic and GABAergic neurons in the three clusters (cluster identity is colour-coded). Note that neurons in Clusters 1 and 3 are REM- and wake-max, respectively. Neurons in Cluster 2 showed weaker brain-state modulation than those in Clusters 1 and 3, but their average activity is wake-max. (**c**) The cell-type composition of each cluster. (**d**) ΔF/F activity at brain state transitions averaged across neurons within each cluster (cluster identity is colour-coded). Grey shading, ±s.e.m. (**e**) Spatial distribution of sleep-wake activity of glutamatergic neurons. Brain-state-dependent activity of each neuron is quantified by the relative distances from the centroids of Cluster 1 (c1 in **a**, REM-max) and Cluster 2 (c2, wake-max), coded by colour. Top panels: spatial organization of neuronal functional properties in two example *VGLUT2-IRES-Cre* mice. Scale bars, 100 μm. Lower panel: spatial organization of all glutamatergic neurons imaged in seven mice. Different symbols indicate cluster identity.

In addition to the difference between glutamatergic and GABAergic cell types (Fig. 2e), individual neurons within each population also exhibited considerable heterogeneity. To further characterize this functional diversity, we performed Principal Components Analysis of the Ca^2+^ activity at brain state transitions (Figs 1e and 2d), followed by k-means clustering (Methods section). We found three distinct clusters (Fig. 3a, Supplementary Fig. 5). The profile of Cluster 1 was characteristic of REM-max neurons (highest activity during REM, Fig. 3b,d). The average activity profiles of Clusters 2 and 3 were both wake-max, although they differed in the amplitude of brain state modulation (Fig. 3b,d). Interestingly, Cluster 1 contained exclusively glutamatergic neurons, Cluster 3 exclusively GABAerigic neurons, and Cluster 2 contained a mixture (Fig. 3c).

In the GABAergic Cluster 3, 29/30 neurons were imaged from a single mouse, and histological examination of the imaging site indicated that these neurons were more dorsal than all other neurons included in this study. All but one of the remaining GABAergic neurons fell in Cluster 2. Glutamatergic neurons in both Clusters 1 and 2 were imaged from multiple mice, so neither group could be explained by a single outlier. To determine the spatial distribution of each functional group, we measured the position of each neuron relative to the centre of the imaging field, the location of which was estimated histologically (Methods section, Supplementary Fig. 1). We found that glutamatergic neurons in Clusters 1 and 2 were mostly segregated along the mediolateral axis, with the REM-max neurons in Cluster 1 located more medially (Fig. 3e, lower panel). Such functional variation along the mediolateral axis was apparent even among neurons imaged in the same imaging session (Fig. 3e, upper panel); furthermore, when we separated the glutamatergic neurons into two groups based on their dorsoventral locations, it is evident in both groups (Supplementary Fig. 6).

## Discussion

Our finding that both GABAergic and glutamatergic neurons are more active during wakefulness and REM sleep than NREM sleep is consistent with previous electrophysiological recordings^18^,^19^,^22^. The activity of both cell types changed before state transitions (Figs 1e and 2d), suggesting that they may contribute to the triggering of brain state changes. As a population, glutamatergic neurons were more active during REM sleep than wakefulness (Fig. 2b–d), consistent with the finding that many neurons in this region expressing *c-fos* after REM sleep rebound are glutamatergic^17^. On the other hand, while in our study no NREM-max glutamatergic neurons were observed, a recent study showed that chemogenetic activation of some dorsal pontine glutamatergic neurons promoted NREM sleep while reducing REM sleep^27^. One difference between the two studies is the targeted location; the brain region studied in the previous study^27^ appears to be slightly more anterior to the region we have imaged. REM-max GABAergic neurons have also been observed in the dorsal pons^18^,^21^. In our study, although all GABAergic neurons belonged to Clusters 2 and 3, which were overall wake-max, within each cluster there was additional functional heterogeneity. In particular, some of the neurons within Cluster 2 were more active during REM sleep than wakefulness (Figs 1d and 3b, above the diagonal line).

Although the precise relationship between firing rates and GCaMP6 Ca^2+^ signals has been measured for some cortical neurons^24^, it remains to be characterized for the dorsal pontine neurons imaged in this study. Compared with electrophysiological recordings, an important limitation of Ca^2+^ imaging is the low temporal resolution. The slow dynamics of the GCaMP6s Ca^2+^ indicator (100–150 ms rise time, ∼1.8 s decay halftime)^24^ does not allow us to determine the precise temporal correlation between the firing of different neurons, the correlation between neuronal activity and phasic events occurring during REM sleep^28^, or burst versus tonic neuronal firing, which may occur preferentially in different brain states^19^.

Nevertheless, compared with juxtacellular recordings followed by immunohistochemical staining, cell-type-specific Ca^2+^ imaging using genetically encoded Ca^2+^ indicators not only greatly increases the rate of data collection but can also reveal spatial organization of the functional properties of each cell type. For instance, although in the cat pharmacological activation of the dorsal pons reliably induces REM sleep^7^,^8^,^9^,^11^,^13^, in rodents this effect is much less consistent^10^,^12^. Our imaging experiments showed that, in the mouse, the REM- and wake-max glutamatergic neurons are located in close proximity (Fig. 3e, Supplementary Fig. 6), which may be why it is difficult to activate REM-max neurons exclusively. On the other hand, the REM- and wake-max glutamatergic neurons are preferentially distributed in the medial and lateral regions, respectively (Fig. 3e). Neurons in the dorsal pons are known to project to multiple targets rostrally in the forebrain and caudally in the medulla and spinal cord^10^,^21^. It is possible that the two groups of glutamatergic neurons identified in our study innervate distinct downstream targets, promoting either the REM or wake state. Our characterization of their spatial distributions will greatly facilitate future efforts to uncover the molecular identity, synaptic connectivity and functional roles of these neurons in sleep–wake control.

## Methods

### Animals and surgery

All procedures were approved by the Animal Care and Use Committee at the University of California, Berkeley. Experiments were performed in adult (2–6 months old), 25–40 g, *ChAT-IRES-Cre* (Jackson Laboratories, B6;129S6-Chat<tm1(cre)Lowl>/J, stock number 006410), *GAD2-IRES-Cre* (Jackson Laboratories, Gad2tm2(cre)Zjh/J, stock number 010802) or *VGLUT2-IRES-Cre* mice (Jackson Laboratories, Slc17a6tm2(cre)Lowl/J, stock number 016963), males and females. The animals were singly housed in a 12/12-h light/dark cycle with lights on from 7:00 to 19:00 with food and water available *ad libitum*.

To image from the dorsal pons across the sleep–wake cycle, mice were implanted with EEG and EMG electrodes as well as a GRIN lens. Mice were anaesthetized with isofluorane (1.5–2%) and placed in a stereotaxic frame (David Kopf Instruments). The skull was exposed and cleaned of connective tissue. We then stereotaxically injected 250–300 nL of AAV1-*FLEX-GCaMP6s* (AAV1.Syn.Flex.GCaMP6s.WPRE.SV40, Penn Vector Core)^24^ into the dorsal pons in or near the LDT (−5.4 mm AP, 0.8 mm ML, 3 and 3.5 mm DV from bregma)^29^ using a borosilicate pipette and Nanoject syringe (Drummond Scientific). A stainless steel headplate was then affixed to the skull with machine screws and dental cement made opaque with carbon powder (Sigma, 484164). A stainless steel screw was implanted 1 mm posterior and 1.5 mm lateral to bregma for EEG recording. A second screw was implanted over the contralateral cerebellum as a reference. EMG electrodes (stainless steel wire loops) were implanted on the neck muscles. All wires were attached to a microstrip connector that was affixed to the skull with dental cement. Finally, at least 45 min after the injection, a microendoscope (GRIN lens, Inscopix, 0.5 mm diameter, 6.1 or 8.4 mm length) was implanted over the injection site (−5.4 mm AP, 0.8 mm ML, 3 and 3.5 mm DV from bregma) using a custom-made holder attached to a stereotaxic frame and covered with a plastic cap. The mice were given one or two doses of buprenorphine (0.05 mg kg^−1^ of body weight, the first before surgery and the second 6–8 h later if needed) and one dose of meloxicam (5 mg kg^−1^ of body weight, before surgery) for pain management. Two to four weeks after the implantation surgery, mice were lightly anaesthetized (1% isofluorane) and head-fixed. The protective cap was removed to expose the GRIN lens and a miniaturized, single-photon, fluorescence microscope (Inscopix) was lowered over the implanted microendoscope until the GCaMP6s fluorescence was visible under illumination with the microscope's LED. The microscope's baseplate was then secured to the skull with dental cement darkened with carbon powder for subsequent attachment of the microscope to the head^24^,^25^. Similar to many other invasive surgery procedures, although some bleeding occurs during the insertion of the lens, by the time we commence imaging (2–4 weeks later) the lens is cleared of blood. Although the procedure is generally more disruptive than traditional single-unit recording, survival rates of the procedure were nearly 100%. After the recovery period, we did not observe any gross behavioural abnormality, and these mice exhibited normal sleep–wake cycles.

### Ca^2+^ imaging and brain state recording

All recordings took place during the light cycle in the mouse's home cage placed within a sound-attenuating chamber. The experimenter was not blind to group allocation. First, mice were briefly anaesthetized with isofluorane to focus and attach the microscope to the baseplate. EEG and EMG were recorded using a RHA2000-EVAL amplifier board (Intan Technologies), filtered between 1 Hz and 7.5 kHz and digitized at 25 kHz. Recordings began at least 30 min after recovery from anaesthesia. Fluorescence data were acquired across multiple sessions to sample each brain state over several hours without excessive bleaching of the calcium indicator. Generally, to maximize the number of imaged cells from each animal we changed the focal plane on different days. In a subset of animals we were able to record from the same cells across 2 days. We imaged 1–3 fields of view per mouse from 16 mice (total of 24 fields of view). Calcium imaging data were acquired using nVista HD software (Inscopix) at 10–15 frames per second under 0.2–0.7 mW illumination.

### Two-photon imaging

Comparisons between two-photon and single-photon Ca^2+^ imaging through the GRIN lens were performed in three *VGLUT2-IRES-Cre* mice. For both two-photon and single-photon imaging sessions, mice were awake and head-fixed.

Two-photon imaging was performed with a Movable Objective Microscope (Sutter Instrument) controlled with ScanImage software^30^. Excitation intensity from a tunable femtosecond laser (Wideband, Tsunami Mode-Locked Ti: Sapphire Laser, Spectra-Physics) was controlled by a Pockels cell (350-80-LA-02; Conoptics). The excitation laser was focused with a 40 × /0.8 numerical aperture infrared objective (LUMPFLEN, Olympus). Fluorescence was collected after a dichroic mirror (650dcxr, Chroma) using a pentagon-style detector into green and red channels with respective emission filters (FF01-510/84-25, Semrock; HQ610/75, Chroma) and photomultiplier tubes (GaAsP H10770PA-40 and multi-task alkali R6357, Hamamatsu). We used an excitation wavelength of 900 nm. Frames of 256 × 256 pixels (∼219 × 219 μm) were acquired at 1.68 Hz.

### Histology

To help localize the position of the GRIN lens (Supplementary Fig. 1), we performed either tyrosine hydroxylase or choline acetyltransferase (ChAT) immunohistochemistry to label the locus coeruleus or the LDT, respectively. Mice were deeply anaesthetized and transcardially perfused with ∼5 ml phosphate-buffered saline (PBS) followed by ∼5 ml 4% wt/vol paraformaldehyde. Imaging locations were identified based on the position of lesions caused by the GRIN lens. The brain was removed and post-fixed overnight in 4% paraformaldehyde, and then placed in a 30% sucrose solution for cryoprotection for 24–48 h. The brainstem was sectioned in 30-μm-thick coronal slices with a cryostat (Leica Microsystems). Slices were washed with PBST (0.2% Triton X-100 in PBS) and incubated for 2 h with blocking buffer (2% normal donkey serum in PBST for ChAT staining or 2% normal goat serum and 50 mg ml^−1^ BSA in PBST for tyrosine hydroxylase staining). The buffer was washed out with PBST and the samples incubated overnight at 4°C with primary antibody (1:200 dilution in blocking buffer, goat anti-ChAT IgG antibody, catalogue no. AB144P, Millipore; or 1:1000 dilution in blocking buffer of polyclonal rabbit anti-tyrosine hydroxylase antibody, ab112, Abcam). Samples were then washed with PBST and incubated for 2 h at room temperature with the secondary antibody (1:250 dilution of Alexa Fluor 594 Donkey Anti-goat IgG antibody or 1:1000 dilution Alexa Fluor 594 Goat Anti-Rabbit IgG antibody, both from Invitrogen)^31^,^32^. Slides were mounted using Vectashield with 4,6-diamidino-2-phenylindole (Vector Labs). Images were acquired with a Hamamatsu nanoZoomer digital pathology (NDP) slide scanner (Hamamatsu NanoZoomer 2.0HT).

### Data analysis

*State classification*. Processing of EEG and EMG data was performed using MATLAB (MathWorks). EEG and EMG signals were spectrally decomposed by fast Fourier transform using a 1-s sliding Hamming window with 0.5-s steps. We then extracted delta (1–5 Hz) and theta (7–10 Hz) EEG power and high-frequency (300–500 Hz) EMG power. Brain state was assessed in 6-s epochs. NREM sleep was identified as epochs with high-delta power, low EMG power, and low theta/delta power ratio. REM sleep was identified as epochs with elevated theta/delta power ratio, low delta power and low EMG power. Finally, wake epochs were identified as periods of elevated EMG activity, high theta/delta power, and low delta power. For analysis of brain state transitions, EEG and EMG activity were manually inspected and adjusted in 2-s epochs to identify transition times more precisely.

*Image processing*. Images were spatially down-sampled by 4-pixel bins using the nVista Viewer (Inscopix). Subsequent processing and analysis of imaging data were performed in MATLAB. To correct for lateral motion of the brain relative to the GRIN lens, we calculated the difference between the image stack and the image stack smoothed with a 20-pixel-radius disk filter (the result is a high-pass filtered image stack). We then selected a high-contrast subregion from the mean projection of the new image stack to be used as a reference^33^. To register each frame to the reference, we calculated the cross-correlation between the reference image and the corresponding subregion of each frame of the high-pass filtered image stack and identified the shift with the maximum correlation. This shift was then applied to the entire frame of the original image stack. For a subset of imaging sessions in which motion was not successfully corrected with the above method, lateral motion was corrected with the open source toolkit advanced normalization tools (ANTs) (refs 34, 35; http://picsl.upenn.edu/software/ants/).

Regions of interest (ROIs) were manually selected from the mean projection of the corrected image stack of the first imaging session of the day. ROIs were aligned across sessions using a semi-automated method whereby the ROI positions from the first session were shifted in the *x* and *y* direction by up to ±5 pixels to maximize the pixel intensity within the ROIs. Alignment was then manually corrected as necessary. In cases where the same ROIs were imaged on multiple days, ROIs selected from the first day were used as the reference for all subsequent imaging sessions and the ROIs were aligned as above.

*Fluorescence analysis*. The pixels within each ROI were averaged to create a fluorescence time series. To remove contamination from out-of-focus neuropil, we extracted the fluorescence from a ring-shaped region with a width of 20 μm from the border of each ROI, excluding pixels in other ROIs, and subtracted this neuropil fluorescence from the ROI fluorescence, scaled by a correction factor (*cf*): *F*_correct_=*F*_raw_*−cf* × *F*_neuropil_. The correction factor was estimated as the ratio between the pixel intensity within a manually selected blood vessel and that of a neighbouring neuropil region devoid of ROIs, subtracting from both an offset value given by the off-lens fluorescence pixel intensity^36^. The resulting correction factor ranged from 0.30 to 0.96, within the range previously reported using similar methods^24^,^37^. In cases where no clear blood vessel was evident, the mean correction factor was used (GAD2: 0.55; VGLUT2: 0.66). Although the exact Ca^2+^ activity trace measured for each ROI was influenced by the correction factor, the brain state dependence of these neurons was insensitive to the correction factor over a wide range of values.

We calculated ΔF/F(t) as (*F*_correct_*(t)*−*F*_0_*(t))/F*_0_*(t)*, where *F*_correct_*(t)* is the neuropil-subtracted fluorescence trace for each ROI. Because we measured the relatively slow fluctuations in fluorescence that occur with brain state changes, there was no clear baseline period in this experimental design. Consequently, *F*_0_*(t)* was estimated as periods when the ROI was relatively inactive^38^. We calculated the variance of *F*_correct_*(t)* for each ROI in 10-s bins and identified inactive epochs as the periods when the variance was below the 20th percentile. Values between the identified inactive periods were linearly interpolated to estimate *F*_0_*(t)*, which was then used to calculate the Δ*F*_correct_*/F*_0_.

*Evaluation of brain state modulation*. To determine each neuron's pattern of activity across the sleep–wake cycle, we averaged *Z*-scored ΔF/F for each state. Because we observed the lowest activity during NREM sleep, and found no neuron that was maximally active during NREM, in Fig. 2e we compared the activity during wakefulness and REM sleep as:


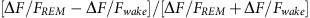


Significant modulation by brain state for each cell type was assessed with a one-way repeated measures ANOVA and two-tailed Tukey's *post hoc* comparisons between all pairs of states. The modulation of each single cell was assessed with a one-way ANOVA and two-tailed Tukey's *post hoc* comparisons.

*Brain state transitions*. To analyse the changes in neuronal activity at brain state transitions, for each neuron we averaged the activity at all individual transitions of each type that contained at least 20 s of the states preceding and following the transition. Using these criteria, 208 neurons were included from 12 mice. We assessed NREM-to-wake, REM-to-wake, wake-to-NREM and NREM-to-REM transitions; direct wake-to-REM or REM-to-NREM transitions occur extremely rarely in normal mice and are thus not considered in this study. The change in activity at the NREM-to-REM transition for GABAergic and glutamatergic neurons was compared with a two-tailed Wilcoxon rank-sum test.

*Cluster analysis of activity patterns at state transitions*. Trial-averaged ΔF/F traces at the four types of transitions (–20 to 20 s in 2-s steps) were concatenated for each neuron (*n*=220 neurons from 5 *GAD2-IRES-Cre*, 7 *VGLUT2-IRES-Cre* and two mice injected with a AAV1-Syn-GCaMP6s, presumably expressed in predominantly glutamatergic and GABAergic neurons). Principal components analysis was performed on the concatenated matrix containing data from both cell types, using singular value decomposition. We then selected the first two principal components (PCs), which accounted for 78.9% of the variance (Supplementary Fig. 5a), and performed k-means clustering based on the Euclidean distance between each cell pair in the two-dimensional PC space. The optimal number of clusters was selected by calculating the Silhouette values for 2–6 clusters and choosing the number associated with the highest average value (Supplementary Fig. 5c).

*Spatial organization of neuronal activity*. To quantify the functional property of each neuron, we calculated its distance from the centroid of Cluster 1 in the 2-D PC space after projecting it onto the axis connecting the centroids of Cluster 1 and Cluster 2 (Fig. 3a). Since Cluster 1 is REM-max, this distance serves as a measure of the similarity between each neuron and a ‘typical' REM-max cell in terms of their activity profiles at state transitions. The anatomical coordinate of each ROI was estimated based on the location of the lesion created by the GRIN lens, identified as described above. The centre of the lesion was assumed to be the centre of the lens, and each neuron's anatomical coordinate was calculated relative to that position. The dorsoventral position of the neuron was defined by the position of the bottom of the lesion, which is likely to be less accurate than the estimate along other dimensions. The single-photon microscope used in this study has a large depth of field, which means that all simultaneously imaged neurons were not necessarily located at the same distance from the lens. The microscope has a 200-μm focal range and the imaged neurons were located 50–250 μm below the bottom surface of the lens.

Because the focusing mechanism of the microscope rotates the objective, we registered each field of view with a reference image and anatomical coordinates from the day of baseplate implantation. To align each field of view with the reference, we found the *x*-*y* shift, image rotation and dilation that maximized the correlation with the reference image^36^.

## Additional information

**How to cite this article:** Cox, J. *et al*. Calcium imaging of sleep-wake related neuronal activity in the dorsal pons. *Nat. Commun.* 7:10763 doi: 10.1038/ncomms10763 (2016).

## Supplementary Material

Supplementary Figures Supplementary Figures 1-6

Supplementary Movie 1 Example imaging session from a *GAD2-IRES-Cre* mouse. Right panels show the EEG power spectrogram, EMG trace, and example ΔF/F traces. Gray shading indicates wakefulness. Speed is 5x. Scale bar: 50 µm

Supplementary Movie 2 Example imaging session from a *VGLUT2-IRES-Cre* mouse. Right panels show the EEG power spectrogram, EMG trace, and example ΔF/F traces. Turquoise shading indicates REM sleep. Speed is 5x. Scale bar: 50 µm.
