# Supplementary material for: Calcium imaging of sleep–wake related neuronal activity in the dorsal pons
Source: Nat Commun. 2016 Feb 25;7:10763. doi: 10.1038/ncomms10763 (PMC4773416; doi:10.1038/ncomms10763)
Supplement: Supplementary Figures — 1-6 [file ncomms10763-s1.pdf]

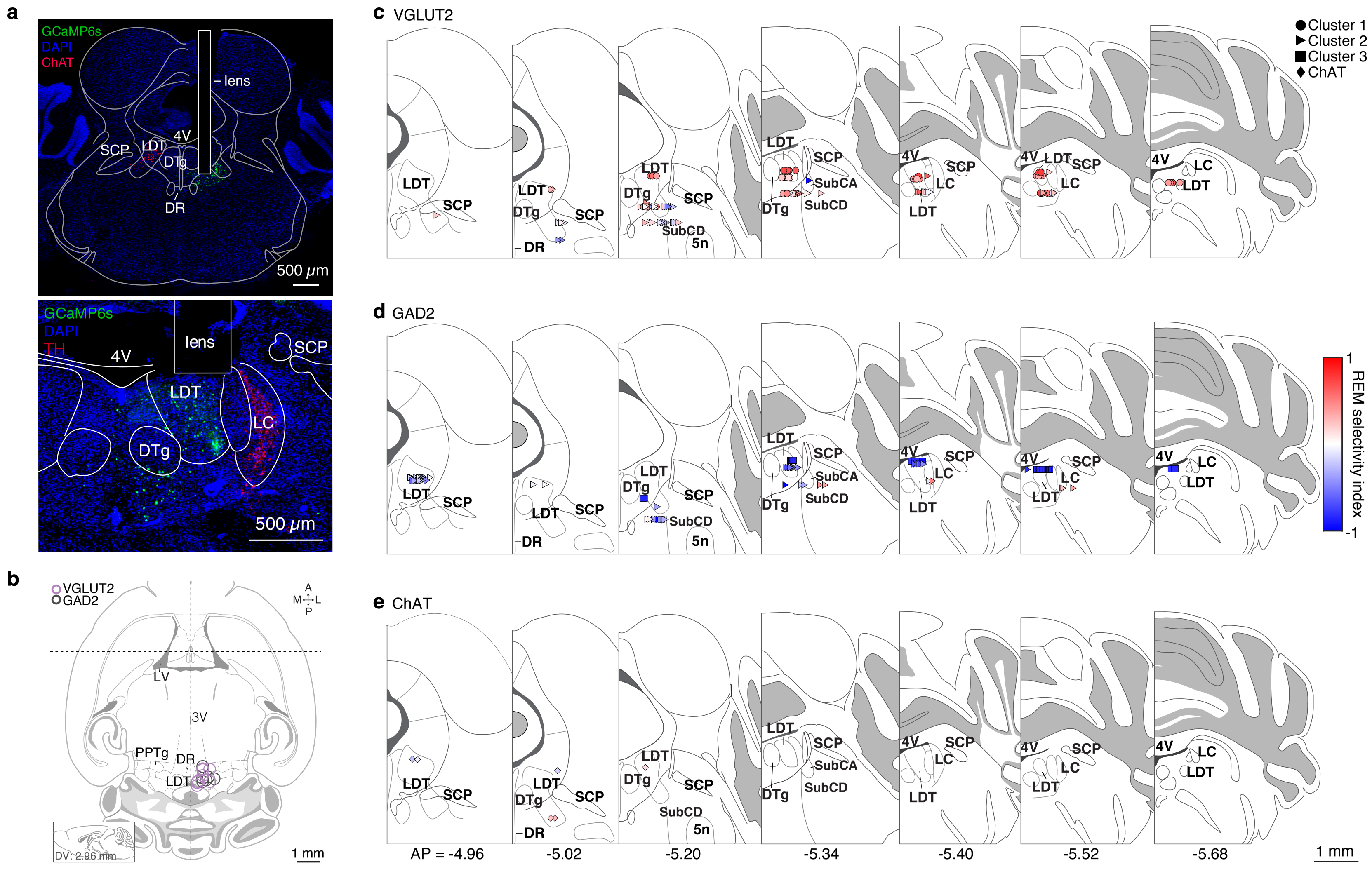
a
GCaMP6s
DAPI
ChAT
lens
4V
SCP
LDT
DTg
DR
500 μm
GCaMP6s
DAPI
TH
4V
lens
SCP
LDT
LC
DTg
500 μm
b
VGLUT2
GAD2
A
M
L
P
LV
3V
PPTg
DR
LDT
DV: 2.96 mm
1 mm
c VGLUT2
d GAD2
e ChAT
LDT
SCP
DTg
DR
SubCA
SubCD
5n
4V
LC
Cluster 1
Cluster 2
Cluster 3
ChAT
REM selectivity index
1
-1
AP = -4.96
-5.02
-5.20
-5.34
-5.40
-5.52
-5.68
1 mm

Supplementary Figure 1 Anatomical positions of individual imaging fields. **(a)** Example imaging locations from a *VGLUT2-IRES-Cre* (top) and *GAD2-IRES-Cre* (bottom) mouse. Anatomical landmarks were identified with tyrosine hydroxylase or choline acetyltransferase immunostaining (red), which are markers for noradrenergic neurons in the locus coeruleus and cholinergic neurons in the LDT, respectively. **(b)** Each circle indicates the location and size of each GRIN lens in a *GAD2-IRES-* (gray) or a *VGLUT2-IRES-Cre* (purple) mouse**.** Horizontal diagram of the brain (depth, 2.96 mm from bregma) was modified from a mouse brain atlas[25]. **(c)** Estimated locations of glutamatergic neurons imaged in this study plotted on a series of coronal atlas sections. Symbols indicate cluster identity (see Fig. 3) and color indicates REM selectivity (see Fig. 2e). **(d)** Similar to **(c)**, for GABAergic neurons. **(e)** Similar to **(c)** for cholinergic neurons. 3V: 3rd ventricle; 4V: 4th ventricle; DTg: dorsal tegmental nucleus; DR: dorsal raphe; LC: locus coeruleus; LDT: laterodorsal tegmental nucleus; LV: lateral ventricle; PPT: posterior pedunculopontine nucleus; SCP: superior cerebellar peduncle; SubCD: subcoeruleus nucleus (dorsal part); SubCA: subcoeruleus nucleus (alpha part).

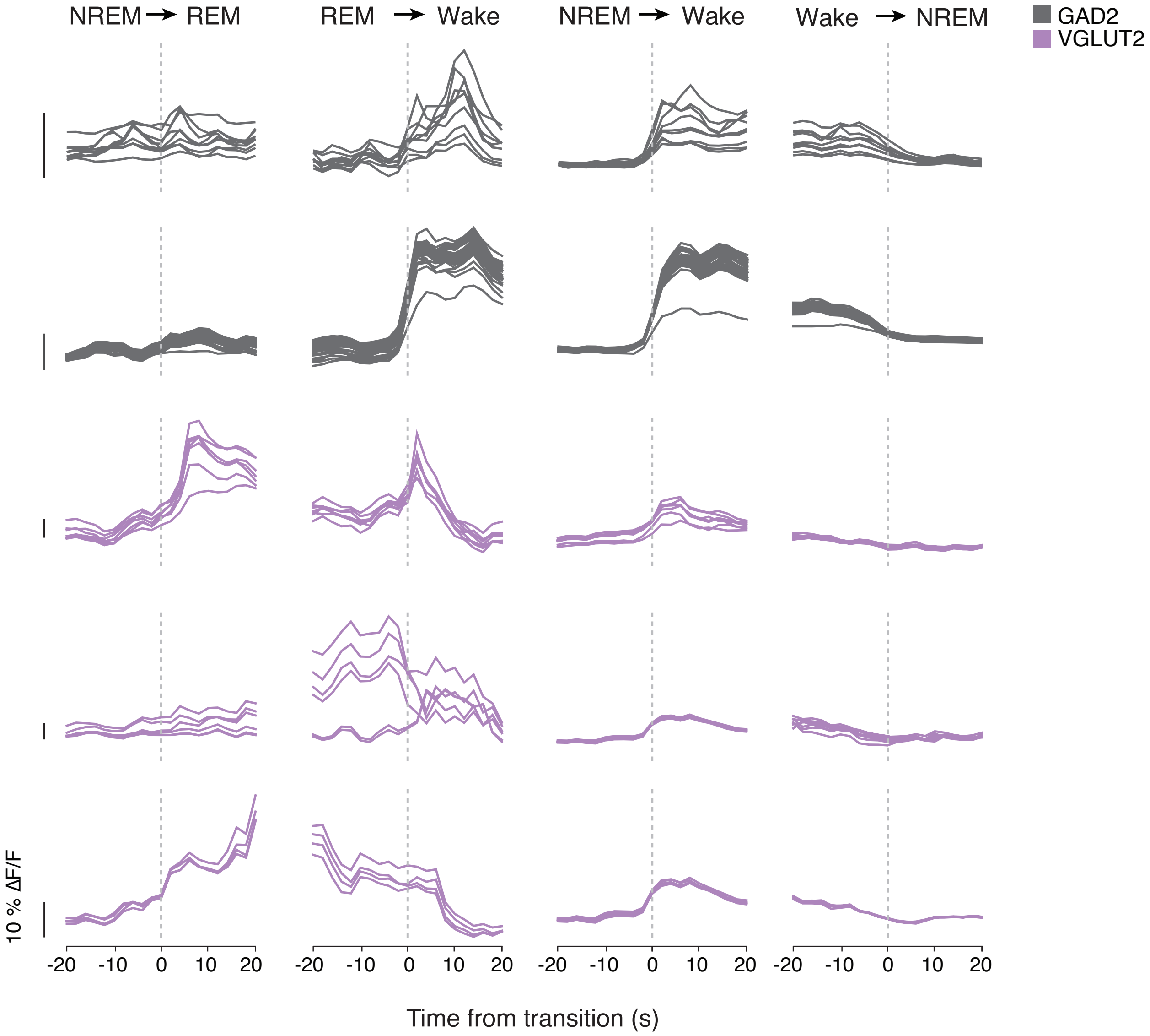

Supplementary Figure 2 Examples of neuronal Ca^{2+} activity during brain state transitions. Shown are ΔF/F traces of GABAergic (gray) and glutamatergic (purple) neurons at brain state transitions (time of transition marked by vertical dashed line). Each trace represents data from a single neuron, averaged across all transitions during the imaging session. Each panel represents data from all simultaneously imaged neurons in a given session.

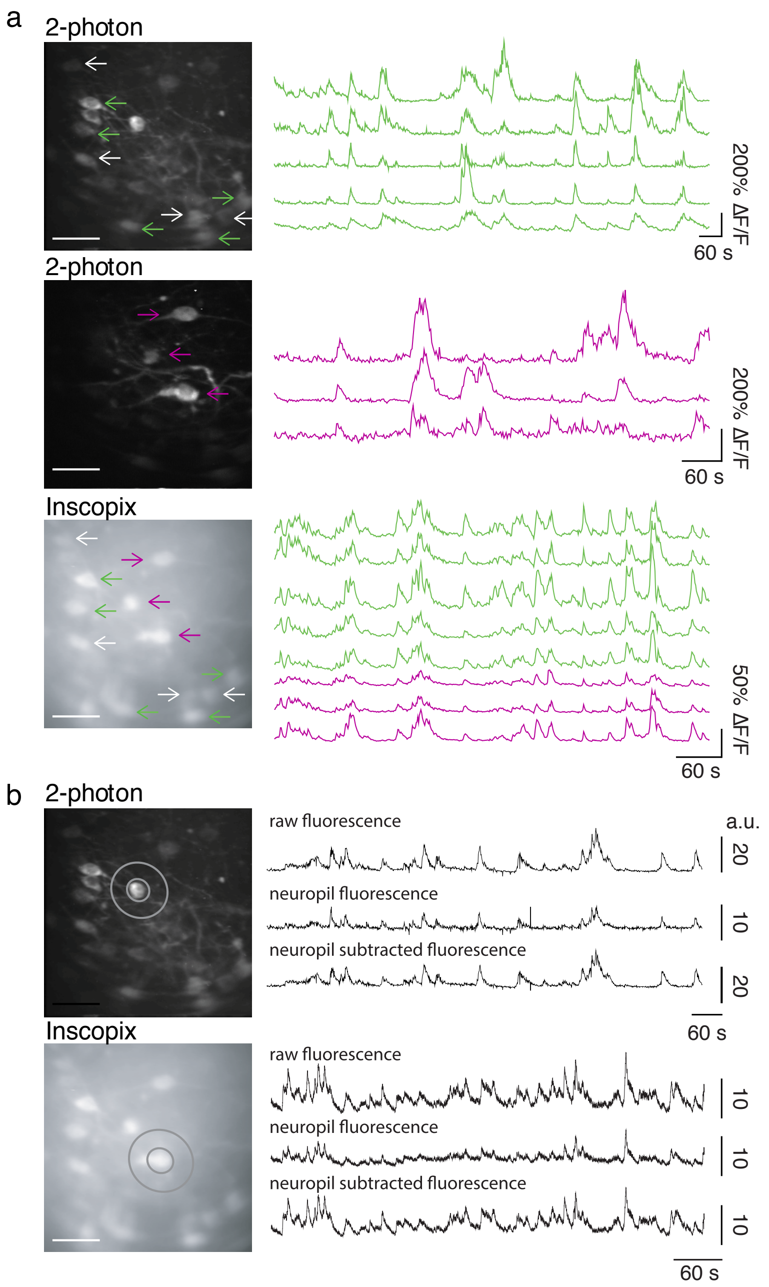

Supplementary Figure 3 Comparison of two-photon and single-photon Ca^{2+} imaging. **(a)** Example imaging fields and ΔF/F traces from neurons imaged with a two-photon microscope (top two panels) and the Inscopix single-photon microscope (bottom panel). Neurons visible with the Inscopix microscope were identifiable at two different imaging depths using a two-photon microscope. Colored arrows indicate neurons detected with both systems and the colors correspond with the two-photon imaging fields. **(b)** Left panels show an example region used for neuropil subtraction (see Methods) for two-photon (top) and single photon (bottom) imaging. The raw fluorescence of the ROI (inner circle), neuropil fluorescence (the region between the two circles), and neuropil-subtracted fluorescence are shown for each neuron. Scale bars: 50 µm

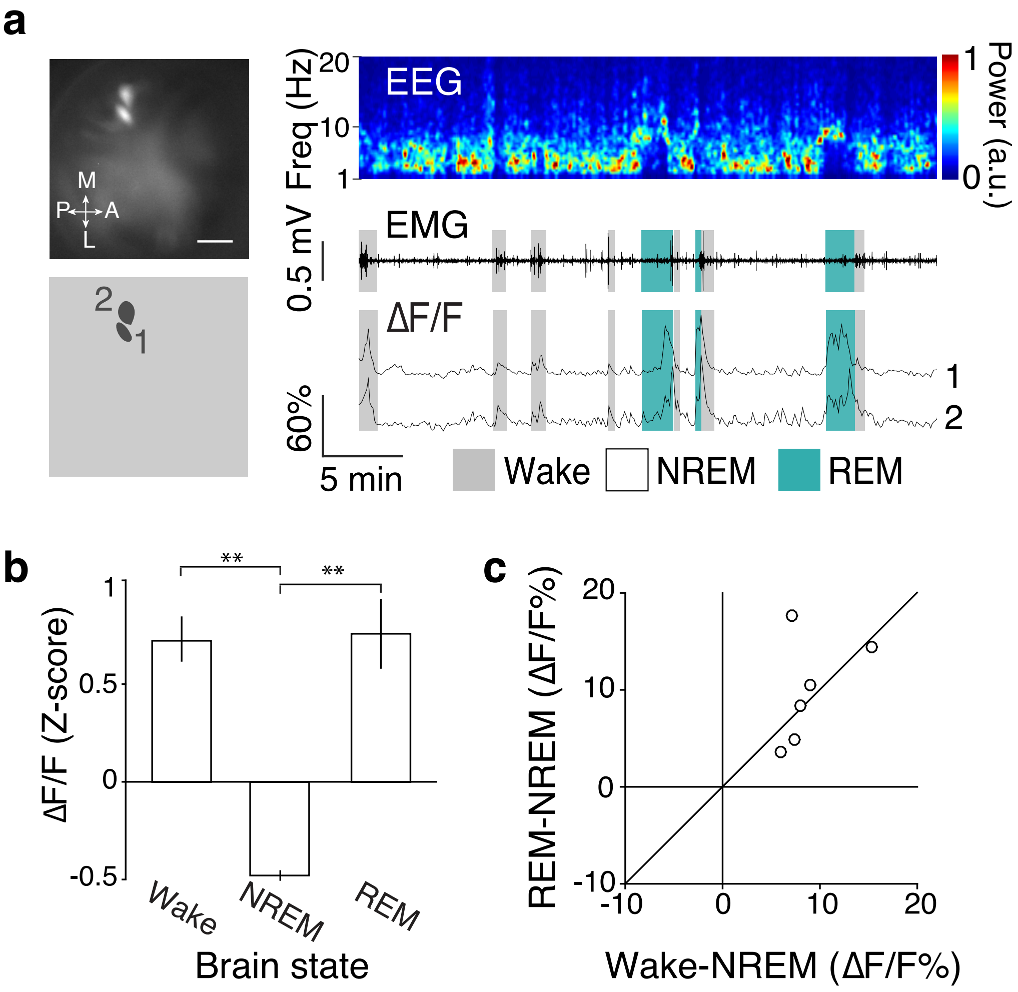

Supplementary Figure 4 Calcium imaging from cholinergic neurons in the dorsal pons. **(a)** Example imaging session from a *ChAT-IRES-Cre* mouse. Left panel: imaging field (upper) and identified regions of interest (lower). Scale bar: 100 µm. Right panels: EEG power spectrogram, EMG trace and ΔF/F traces. Brain states are indicated by colored shading. **(b)** Average Z-scored ΔF/F activity during wakefulness, NREM and REM sleep (n = 6). $F_{(2,5)}$ = 24.46, $P = 1.4 \times 10^{-4}$, one-way repeated measures ANOVA; ** $P < 0.001$, two-tailed Tukey's post-hoc comparison. **(c)** Difference between REM and NREM activity vs. difference between wake and NREM activity. Each symbol represents one neuron (n = 6).

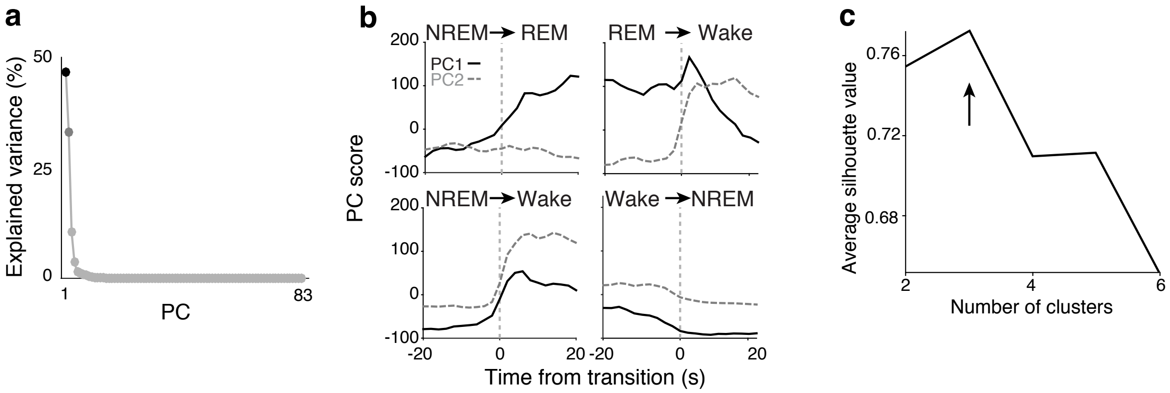

Supplementary Figure 5 Principal Components Analysis and k-means clustering of brain-state-related neuronal activity. **(a)** Percent of the variance explained by each principal component (PC). The first two PCs together explain 78.9% of the variance. **(b)** The first two PCs, each representing the activity profile at each of the four brain state transitions. **(c)** Average Silhouette value as a function of the number of clusters. The optimal number of clusters (3) was chosen as the maximal average silhouette value (arrow).

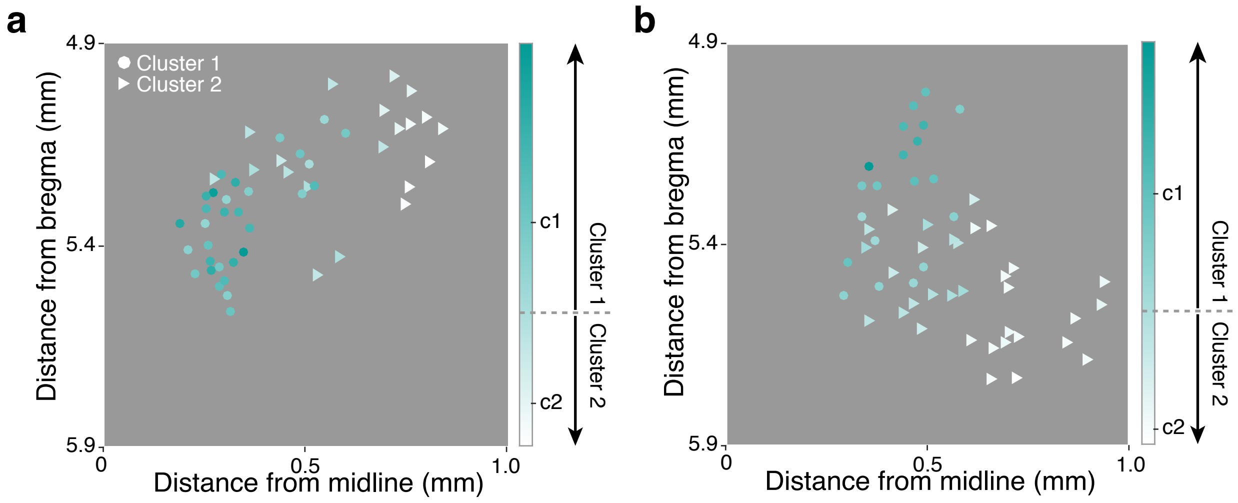

Supplementary Figure 6 Spatial organization of dorsally and ventrally positioned glutamatergic neurons. The brain-state-dependent activity of each neuron is quantified by the relative distances from the centroids of Cluster 1 (c1 in Fig. 3a, REM-max) and Cluster 2 (c2, wake-max), coded by color. (**a**) Spatial organization of dorsally positioned glutamatergic neurons, located between 2.3 – 3 mm from the surface of the brain. (**b**) Spatial organization of ventrally positioned glutamatergic neurons, located between 3 – 3.3 mm from the surface of the brain.
